# Biomass-Derived Activated Porous Carbon from Foxtail Millet Husk to Utilizing High-Performance Symmetric Supercapacitor Applications

**DOI:** 10.3390/nano15080575

**Published:** 2025-04-10

**Authors:** Perumal Rajivgandhi, Vediyappan Thirumal, Alagan Sekar, Jinho Kim

**Affiliations:** 1 Department of Chemistry, Nehru Memorial College (Affiliated to Bharathidasan University), Puthanampatti, Trichy 621 007, India; rajlibniz@gmail.com; 2 Department of Mechanical Engineering, Yeungnam University, Gyeongsan-si 38541, Republic of Korea; thirumalvisnu@gmail.com

**Keywords:** foxtail millet husk, biomass, activated carbon, energy storage, supercapacitor

## Abstract

This study successfully demonstrates the synthesis of foxtail millet carbon-activated (FMCA) materials using a two-step carbonization process from foxtail millet husk (FMH). The pre-carbonized biomass-derived millet husk was chemically activated with KOH at 500 °C and subsequently carbonized in an inert argon atmosphere at 800 °C in a tubular furnace. XRD analysis revealed a diffraction peak at 2θ = 23.67°, corresponding to the (002) plane, indicating the presence of graphitic structures. The Raman analysis of FMCA materials showed an intensity ratio (I_G_/I_D_) of 1.13, signifying enhanced graphitic ordering and structural stability. The as-prepared FMC and FMCA electrode materials demonstrate efficient charge storage electrochemical symmetric devices. Electrochemical analysis revealed the charge–discharge curves and a specific capacitance of Csp (FMC//FMC) 55.47 F/g and (FMCA//FMCA) 82.94 F/g at 0.5 A/g. Additionally, the FMCA//FMCA symmetric device exhibits superior performance with a higher capacity retention of 94.89% over 5000 cycles. The results confirm the suitability of FMCA for energy storage applications, particularly in electrochemical double-layer capacitors (EDLCs), making it a promising material for next-generation supercapacitors.

## 1. Introduction

Energy storage technologies are rapidly evolving, particularly with advancements in supercapacitors, also known as ultracapacitors, also known as electric double-layer capacitors, which rely on the rapid and reversible adsorption and desorption of ions at the electrode–electrolyte interface to store and release energy [1,2,3]. Recent developments in symmetrical carbon-based materials have demonstrated significant improvements in cycle life and performance for both electric double-layer capacitors (EDLC) and pseudo-capacitance, leading to enhanced efficiency in energy storage systems [4,5]. In recent years, the utilization of metal oxide nanomaterials has unveiled favorable redox behavior, contributing to long-lasting performance with minimal capacity loss even after thousands of charge–discharge cycles [6,7]. Meanwhile, biomass hard carbon derived from biomass and activated carbon materials have emerged as promising alternatives due to their scalable production, high porosity, and exceptional surface area [8]. These bio-derived carbon-based hard nanomaterials are essential in optimizing charge storage mechanisms, significantly improving both the efficiency and lifespan of supercapacitor devices [9]. By leveraging their unique structural properties and high conductivity, they facilitate faster charge and discharge cycles while maintaining stable performance over time. This results in more durable, energy-efficient supercapacitors, contributing to the development of advanced energy storage technologies with a lower environmental impact [10].

In recent years, agro-waste bio-derived activated carbon is a versatile material that has garnered significant attention in the field of energy storage due to its high surface area and porous structure [11]. These porous and higher surface properties have the capacity to store energy through the formation of electric double layers at the carbon–electrolyte interface [12]. The production of activated carbon from waste materials offers the use of acid- and alkaline-based surface modifications for a sustainable and cost-effective approach [13,14]. It is a compelling choice for various energy conversion and storage applications, particularly supercapacitors, resulting in improved energy density and power density in supercapacitor devices [15]. Various types of activated carbons, including those commercially available, typically have lower porosity and surface activation than biomass-derived carbons [16,17]. Biomass-derived carbons exhibit superior surface reaction capabilities and increased porosity, which promote enhanced ionic storage when activated with alkaline-based agents such as NaOH, H_3_PO_4_, FeCl_3_, and ZnCl_2_ [18,19]. Biomass waste products, often discarded, can be repurposed through large-scale chemical activation methods to produce high-performance activated carbons. Among various techniques, alkaline activation, particularly using KOH, combined with pretreatment and thermal pyrolysis (typically between 600 and 1200 °C), significantly enhances surface area and porosity [20,21,22,23].

In addition, alkaline activation converts agricultural and other waste materials into bio-derived activated carbon materials, offering a sustainable approach for electrode fabrication [24]. Such bio-derived carbons are highly promising electrode active materials for application in symmetric supercapacitors, batteries, and energy conversion systems due to their low cost, environmental benefits, and exceptional electrochemical properties [25,26,27,28]. Utilizing agro-waste for carbon production thus provides a dual advantage: waste management and the development of efficient energy storage materials [29]. There are several biomass agro-waste products utilized with a wide range of applications [30,31,32,33,34,35,36].

Among them, foxtail millet husk (FMH) is a rich source of various bioactive compounds that are rich in fiber content. It contains significant amounts of phenolic compounds, flavonoids, and other phytochemicals that can impact its thermal stability and reactiveness during activation [37,38]. Its major constituents include cellulose, hemicellulose, and lignin of the plant-available content of silica/silicon [39]. After KOH activation, chemical activation with KOH allows for the transformation of foxtail millet husk into highly porous activated carbon. In this process, KOH not only serves as a chemical agent that enhances pore formation but also increases the total surface area of the resultant carbon material, often exceeding 1200 m^2^/g [40]. The thermal carbonization process typically occurs at high temperatures, around 600 to 800 °C, where biomass undergoes pyrolysis to produce biochar, thus concentrating carbon content [41].

Foxtail millet husk (FMH) is one such agro-waste product that is reported as having antioxidant, acetylcholinesterase, and α-glucosidase inhibitory activities [42]. FMH waste is converted into energy in eco-friendly supercapacitor applications. Foxtail millet husk (FMH), as ground millet seeds, was once discarded as agro-biomass, but nowadays it is utilized by researchers as next-generation supercapacitors. The thermally activated carbon that transforms FMH into a high-performance electrode material redefines sustainability in energy storage. From FMH, activated bio-derived carbon finds new life in clean energy applications. The activated carbon has good physicochemical properties essential for effective energy storage capabilities [43] and thermoplastic composites [44]. Among them, foxtail millet husk-derived biomass provides valuable materials for energy storage with green bio-energy technologies [45], and chemical activation with potassium hydroxide (KOH) and thermal carbonization, which can significantly enhance its physico-chemical properties for uses in energy storage, particularly in supercapacitors [46,47].

Specifically, this study highlights the synthesis of highly porous activated carbon derived from agro-waste Fox millet husk (FMH) using KOH-assisted chemical activation [48]. Different conventional biomass carbonization techniques without activating agents typically yield a low surface area and limited porosity, whereas our method significantly enhances the surface area and pore structure, promoting improved ionic transport and electrochemical performance [48,49]. This KOH-activated FMH carbon demonstrates superior specific capacitance, energy density, and excellent cycling stability compared to non-activated and traditionally derived carbon materials [50]. These improvements underscore the potential of FMH as a sustainable, cost-effective, and scalable precursor for high-performance supercapacitor electrodes [51].

This work first reported pure FMC, and then chemically activated with carbonized activated FMCA materials, for KOH activation pre-carbonized materials at 500 °C and, subsequently, then carbonized in an Argon inert atmosphere at 800 °C in a tubular furnace. These milled husk biomass-derived activated carbons were analyzed before and after activation of KOH, using FE-SEM and FE-TEM surface morphological conformation with a porous nature with thick-platelet micro-structural particles. In addition, as-prepared FMC and FMCA materials applied great promise as electrode materials due to their high surface area, good electrical conductivity, and excellent chemical stability. Further, the cyclic volumetry (CV) curves and charge–discharge performance had good responses for electrochemical electric double-layer capacitor (EEDLC) properties. The electrochemical potentiostatic impedance spectra represent a lower charge-transfer resistance. The electrode materials’ cycling stability had high performance over 5000 cycles, which is good performance for supercapacitor applications.

## 2. Materials and Methods

The waste that had been physically adsorbed on the surface of foxtail millet husk (FTH) was sourced locally from Valapadi in the Salem district of Tamil Nadu, India. The husk of foxtail millet was removed by repeatedly grinding the seeds and rinsing them thoroughly with double-distilled water. The processed samples were then dried in a hot air oven at 100 °C. The collected FTH was thoroughly milled with a home kitchen blender to make a fine powder, before being pre-carbonized in an Ar environment at 500 °C in a tubular furnace. Then, using a mortar and pestle, the pre-carbonized materials were thoroughly pulverized. They were then carbonized in an Ar atmosphere at 800 °C. To further eliminate the metal particles, the collected sample was treated with 6N HCL acid solution. The sample was dried following multiple water washes. In order to eliminate the undesirable Si that reduces the material’s conductivity, the dried FMC sample was treated with 40% HF. The sample was additionally cleansed with DD water to achieve a neutral PH and dried at 100 °C in an oven for 8 h. The sample that was produced by the foregoing procedure was given the name FMC.

**Figure 1:**
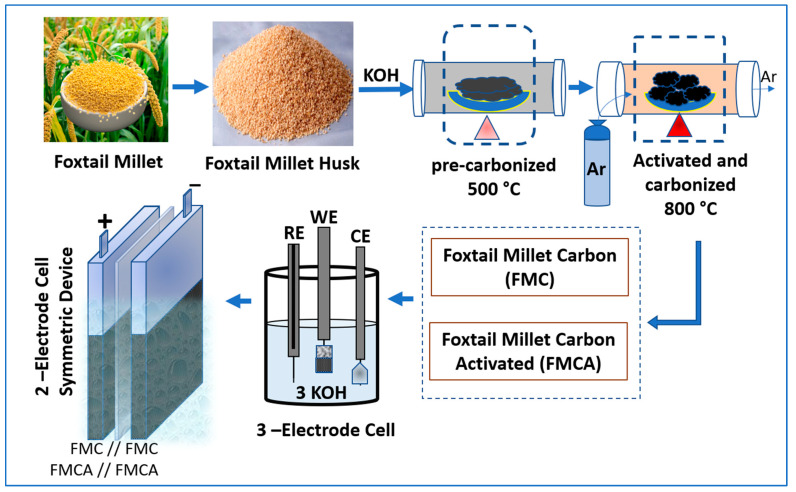
A schematic showing the foxtail millet husk photographs and KOH activation process. Illustration of the three-electrode cell configuration and also the device’s working electrode with two-electrode-cell setup.

In addition, KOH activation was applied to one portion of the FMC sample as follows. Agate mortar and pestle were used to combine the sample and KOH in a 1:2 weight ratio to create ether. The hygroscopic properties of KOH made it possible to combine it inseparably with FMC, which improved the pores and produced more active sites. This suspension was prepared and left undisturbed for 48 h. It was then washed with a lot of DD water to bring the PH balance back to neutral, then dried for 8 h at 100 °C in the oven. The substance was successfully obtained and given the name FMCA. A detailed explanation schematic (Figure 1) is given to understand the graphical abstract for foxtail millet husk activated biomass porous carbon for electrochemical symmetric supercapacitor device applications.

### 2.1. Materials Characterization

The characterization of the as-prepared FMC and the activated FMCA carbon materials was conducted to confirm their structural and morphological properties. The phase composition of the samples was analyzed using X-ray powder diffraction (XRD) with an “X’Pert PRO PAN-Analytical” system (Worcestershire, UK), operating at 40 kV and 30 mA with a Cu-Kα radiation source (λ = 1.5406 Å). The diffraction patterns were recorded in the 2θ range of 10° to 80°. Laser Raman spectroscopy was performed using an XPLORA Plus (HORIBA Scientific, Horiba Jobin Yvon, France) at room temperature, employing a 532 nm Nd:YAG laser with an exposure time of 100 s and an operating power of 10 mW. The surface morphologies of FMC and FMCA were examined using a TESCAN VEGA3 Field Emission Scanning Electron Microscope (FE-SEM), coupled with a Bruker Energy Dispersive X-ray Spectroscopy (EDS) system (Brno, Czech Republic), operated at an accelerating voltage of 15 kV. Further nanoscale structural analysis was conducted using Field Emission Transmission Electron Microscopy (FE-TEM) with an “FEI-TECNAI TF-20” (Hillsboro, OR, USA) at an accelerating voltage of 200 kV. The specific surface area, pore volume, and pore size distribution of the samples were determined using the Quantachrome NOVA 2200E BET Surface Area Analyzer (Autosorb-1-C-8) (Boynton Beach, FL, USA), which is a fully automated system capable of measuring BET surface areas as low as 0.1 m^2^.

### 2.2. Electrode Fabrications and Electrochemical Characterization

Foxtail millet husk-derived activated carbon (FMC and FMCA) was utilized as the active material for supercapacitor electrode fabrication. The electrode slurry was prepared by mixing 80 wt% of the active material (FMC or FMCA), 10 wt% conductive carbon (Super P), and 10 wt% polyvinylidene fluoride (PVDF) binder in an N-methyl-2-pyrrolidone (NMP) solvent to form a homogeneous paste. The cleaned pure Ni-foam substrate, measuring 2 cm × 1 cm, was manually coated with the slurry using a hand brush, ensuring a uniform coating over a 1 cm × 1 cm active area. The coated electrodes were dried at 80 °C for 12 h under vacuum to remove residual solvents and enhance adhesion. For electrochemical characterization, a three-electrode system was employed, using Ag/AgCl as the reference electrode and platinum as the counter electrode in a 3M KOH aqueous electrolyte. The working potential range was maintained between 0.0 V and −0.8 V. Additionally, a two-electrode symmetric supercapacitor device was assembled using FMC//FMC and FMCA//FMCA configurations as both anode and cathode. Whatman glass fiber filter paper was used as a separator, pre-soaked in 3M KOH electrolyte. Finally, both electrodes and the separator were carefully stacked and enclosed within an alumina-plastic shielded pouch cell, with a few electrolyte drops added to ensure optimal ion transport.

Cyclic voltammetry (CV) and galvanostatic charge–discharge (GCD) measurements were conducted in a potential window of 0.0 V to 1.0 V to evaluate the electrochemical performance. Electrochemical impedance spectroscopy (EIS) was performed to analyze the charge transfer resistance and ion diffusion characteristics of the assembled device. The specific capacitance was determined from the GCD curves, providing insights into the charge storage capability and energy efficiency of the biomass-derived carbon electrodes. The specific active mass loading for electrochemical testing ranges from 1.3 mg to 2.8 mg for three-electrode systems and two-electrode symmetric device electrodes.

The specific capacitance (Csp, F/g) of the symmetric supercapacitors, derived from GCD tests, is calculated using Equation (1):

(1) $$C_{\mathrm{sp}}=\frac{I\times \Delta t}{m\times \Delta V}$$

where I is the discharge current (A), t is the discharge time (s), ΔV represents the voltage window between V_max_ and V_min_, and m (g) is the total mass of the active materials in both the anode and cathode.

## 3. Results and Discussion

### 3.1. X-Ray Diffraction Analysis

**Figure 2:**
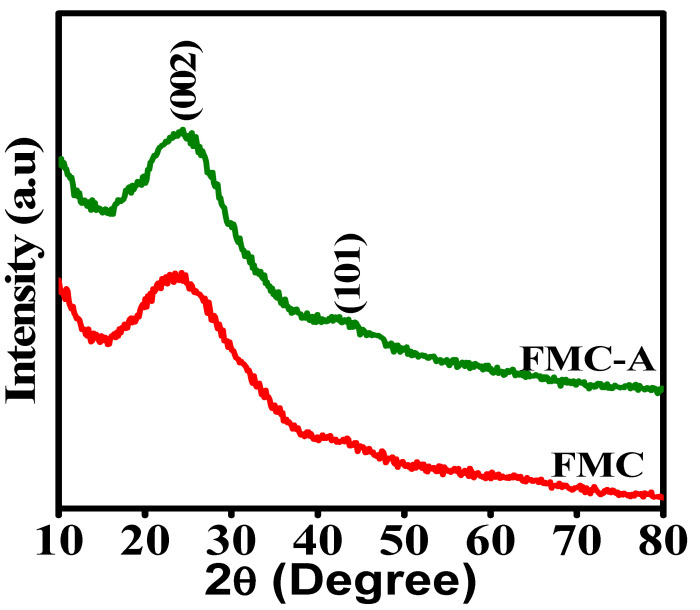
XRD patterns for FMC and FMCA materials.

The X-ray diffraction (XRD) analysis was conducted on carbon materials derived from foxtail millet husk, both before and after chemical and thermal carbonization shown in Figure 2. The pure activated biomass-derived carbon (FMC) exhibited a characteristic diffraction peak at 2θ = 23.67°, corresponding to the (002) plane. After KOH activation and subsequent biomass carbonization, the (002) reflection shifted slightly to 2θ = 24.62°, indicating the formation of an sp^2^-hybridized hexagonal carbon crystal structure. This slight shift in the broad peak, originally centered around 2θ = 23–25°, suggests that KOH activation led to an improved carbonization process, enhancing the structural ordering of the carbon matrix. The well-carbonized nature, along with the high carbon content, is further supported by the minimal presence of amorphous SiO_2_. Additionally, a minor peak observed at 2θ = 42.92° corresponds to the (101) plane, which is commonly present in both materials. Finally, the XRD patterns confirm the well-crystalline nature of FMC and FMCA (thermally activated carbon samples), both before and after KOH activation, as evidenced by the broad yet distinct diffraction peaks.

### 3.2. RAMAN Analysis

**Figure 3:**
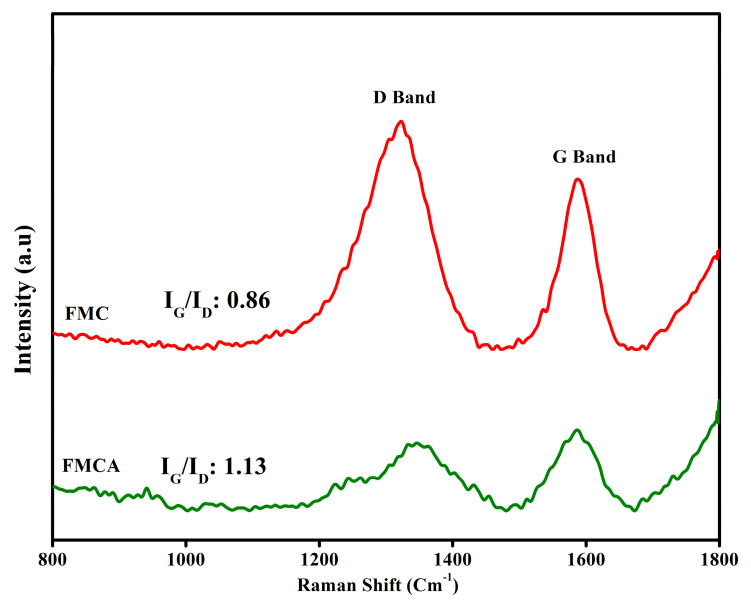
Raman spectra of FMC and FMCA materials.

In Figure 3, Raman analysis of foxtail millet husk-derived carbon and its activated carbon counterpart reveals characteristic fingerprint regions associated with structural disorder and graphitic ordering. The disorder-related D-band, corresponding to sp^3^-hybridized carbon, appears in the range of 1300–1350 cm^−1^, while the graphitic (sp^2^) carbon G-band is observed in the 1500–1600 cm^−1^ region. The Raman spectrum of biomass-derived FMC materials shows D- and G-band peaks at 1321.71 cm^−1^ and 1588.87 cm^−1^, respectively, with an intensity ratio (I_G_/I_D_) of 0.86, indicating a lower degree of graphitization. In comparison, the activated FMCA sample exhibits D- and G-band peaks at 1349.10 cm^−1^ and 1588.21 cm^−1^, respectively, with an increased intensity ratio (I_G_/I_D_) of 1.13, signifying improved graphitic ordering. While both materials exhibit well-graphitized G-band characteristics, the partial presence of the D-band in the samples is attributed to the inherent presence of SiO_2_ in the husk and the hydroxyl functional groups introduced during KOH activation. Overall, Raman analysis confirms that FMCA, derived from biomass, demonstrates enhanced sp^2^-hybridized C=C bonding, making it a well-activated carbon material.

### 3.3. FE-SEM Surface Morphological Analysis

**Figure 4:**
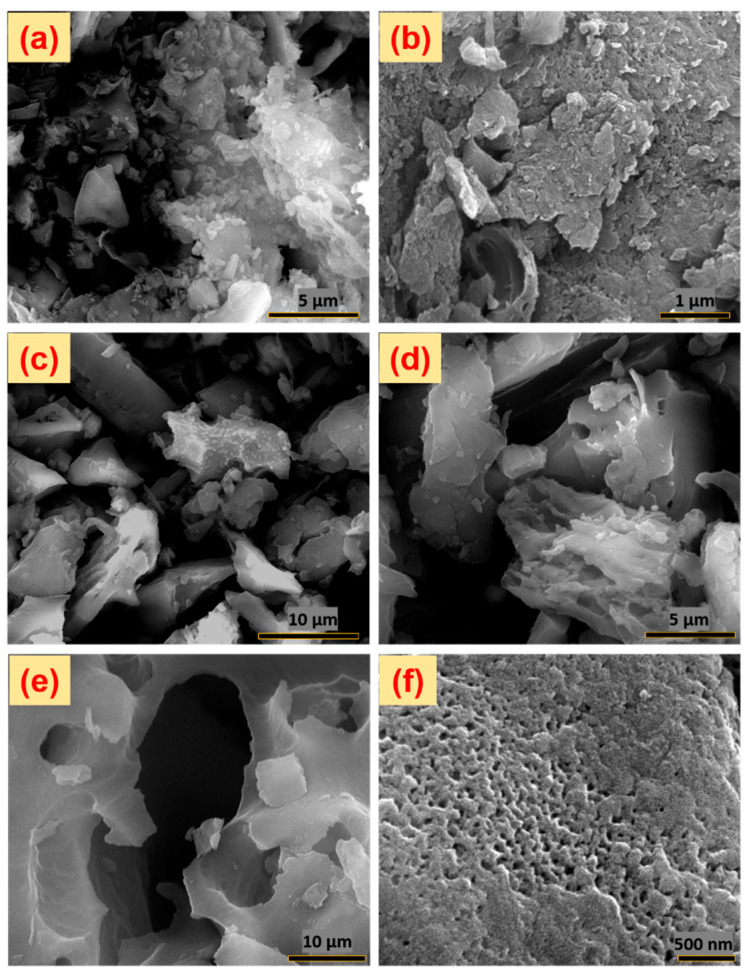
FE-SEM surface micro-structural analysis for (**a**,**b**) FMC and (**c**–**f**) FMCA materials.

The FE-SEM analysis of foxtail millet husk-derived carbon and its activated form provides detailed surface morphological characterization at different magnifications, as shown in Figure 4a–f. The SEM images of foxtail millet carbon without KOH activation display bulk platelet-like structures with uneven particle distribution. In contrast, after activation (Figure 4c,d), the sample exhibits a more refined morphology with fewer, well-defined micron-sized activated carbon particles. Higher magnification images (Figure 4e,f) reveal a porous structure with numerous macro- and micropores, forming a cave-like 3D morphology. The depth-resolved surface images further confirm the presence of well-developed porosity, which enhances ion adsorption and desorption processes. This improved porous architecture, achieved through KOH activation and thermal carbonization, makes FMCA a promising material for electrochemical energy storage applications.

### 3.4. FE-SEM, EDS Elemental Mapping Analysis

**Figure 5:**
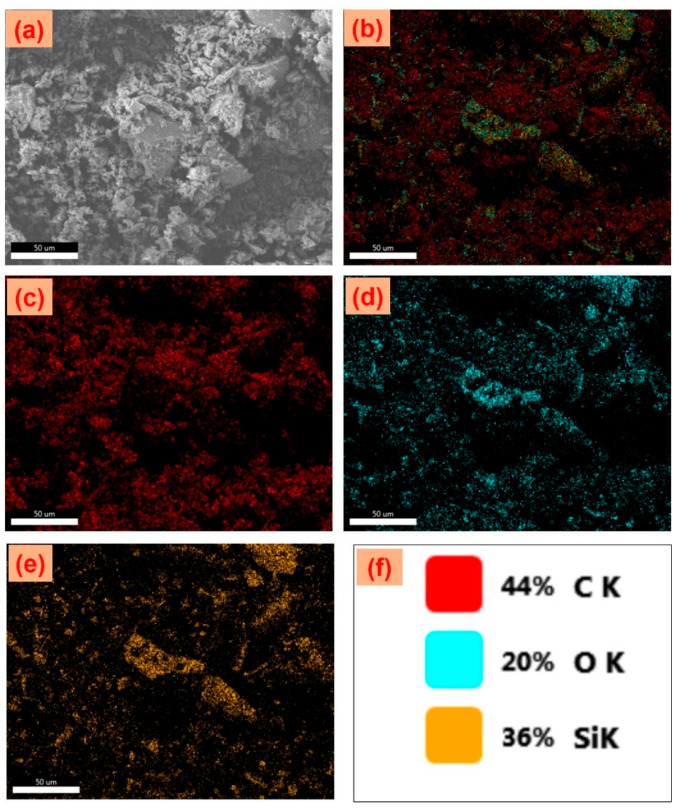
(**a**) FE-SEM image with EDS elemental mapping of FMCA material, (**b**) overall element distribution, and individual maps of (**c**) carbon (C), (**d**) oxygen (O), (**e**) silicon (Si), and (**f**) atomic percentage distribution (color-coded).

The FE-SEM analysis of foxtail millet husk-derived carbon reveals its surface morphology at lower magnification, as shown in Figure 5a. The corresponding FE-SEM-EDS mapping confirms the elemental distribution of C K, O K, and Si K in the analyzed region. Figure 5b presents the overall elemental mapping, clearly displaying a uniform distribution of carbon, oxygen, and silicon across the surface. Additionally, individual element distribution maps in Figure 5c–e further illustrate the distinct spatial dispersion of C K, O K, and Si K, with fine dot-like mapping patterns. The final surface elemental mapping (Figure 5f) provides a weight ratio representation of these elements, with color-coded maps highlighting their respective distributions and also the atomic ration values table (Table S1) provided in Supporting Information (SI). This EDS mapping analysis confirms the presence of FMCA particles, demonstrating their well-developed structure due to effective thermal activation and carbonization processes.

### 3.5. FE-TEM Analysis

**Figure 6:**
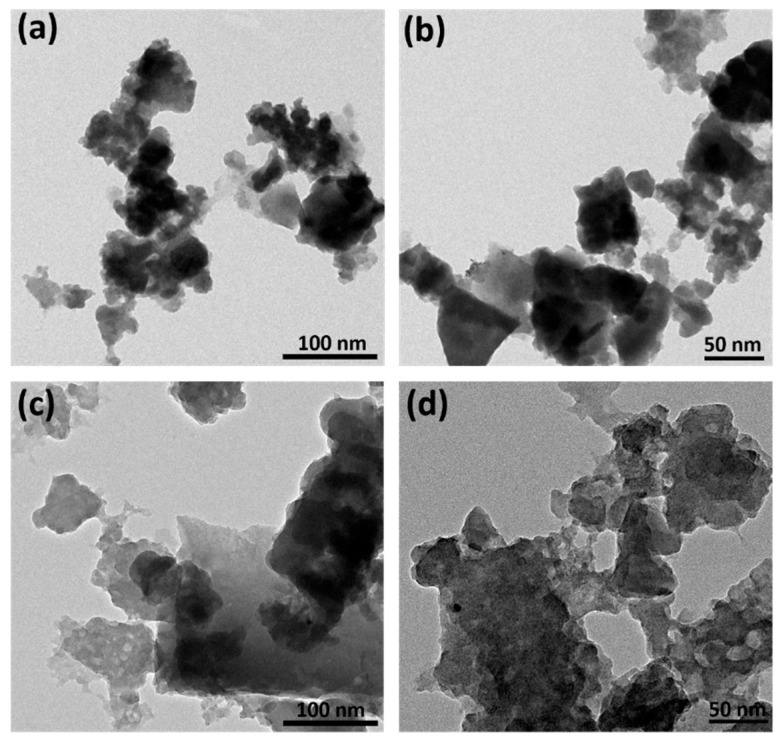
FE-TEM surface micro-structural analysis for (**a**,**b**) FMC and (**c**,**d**) FMCA material.

The FE-TEM analysis of foxtail millet husk (FMH)-derived carbon and its KOH-activated counterpart provides a detailed nanoscale morphological characterization at various magnifications, as shown in Figure 6a–d. The TEM images of foxtail millet-derived carbon before activation display thick, bulk particles with uneven distribution. In contrast, after KOH activation (Figure 6c,d), the FMCA sample exhibits a well-developed micro-porous structure, with visible surface porosity at lower magnifications. Further magnified images (Figure 6d) reveal a highly porous morphology with numerous macro- and micropores distributed across the surface. The KOH-activated FMCA carbon demonstrates an extensively developed surface area, indicating higher degrees of carbonization, which enhances its potential for increased ionic storage sites. This improved porosity and surface structure make FMCA a promising candidate for large-scale biomass-derived carbon production, particularly for electrochemical energy storage applications.

### 3.6. BET Surface Analysis

**Figure 7:**
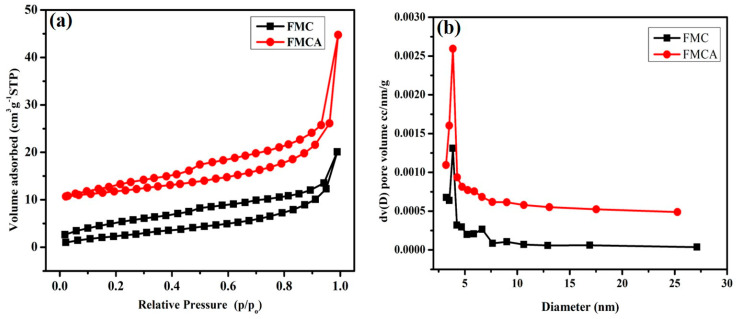
BET analysis: (**a**) Nitrogen adsorption–desorption isotherms plots; (**b**) BJH plots of the pore size distribution.

The nitrogen adsorption–desorption isotherms of the activated carbons, as shown in Figure 7a, provide a comparative analysis of FMC and FMCA, both derived from foxtail millet husk biomass. The Brunauer–Emmett–Teller (BET) surface area measurements indicate that FMC carbon has a surface area of 9.515 m^2^/g, while FMCA carbon exhibits a significantly higher surface area of 14.946 m^2^/g, with a pore volume of 0.024 cc/g. The BET pore size distribution, shown in Figure 7b, reveals that FMCA carbon has a pore radius (Dv(r)) of 19.320 Å, indicating a combination of microporous and mesoporous structures. The isotherms of FMC show significant uptake at low relative pressures, corresponding to adsorption within limited micropores. In contrast, FMCA carbon exhibits type IV isotherms with a type H4 hysteresis loop at intermediate and high relative pressures, characteristic of mesoporous and nanoporous carbon. This enhanced porosity is likely attributed to the KOH activation and thermal carbonization process, which contribute to the formation of well-developed pore structures. The high surface area and porous architecture of FMCA carbon make it a promising candidate for electrochemical energy storage applications, particularly for ion adsorption and charge storage in biomass-derived carbon materials.

## 4. Electrochemical Analysis

**Figure 8:**
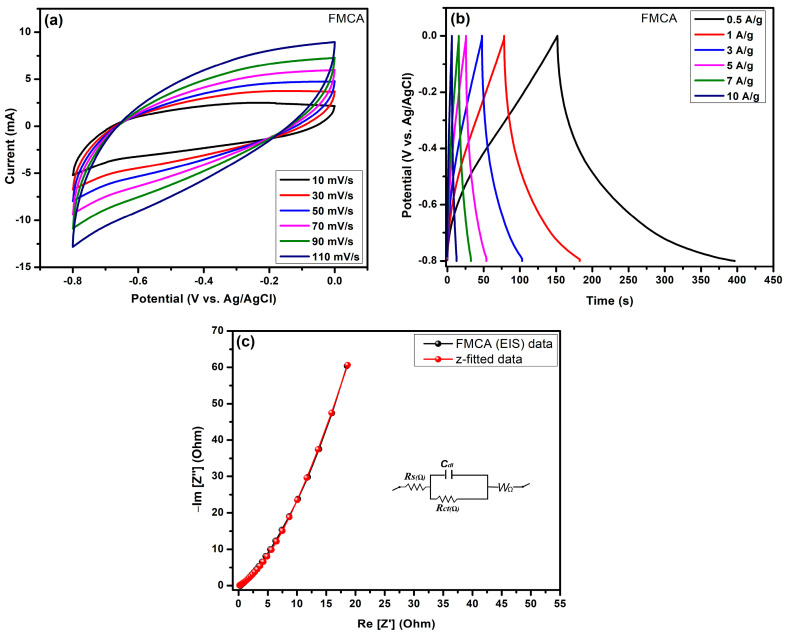
(**a**–**c**) Displays the electrochemical performance for FMCA activated biomass carbon electrode; (**a**) Cyclic Voltammetry (CV) curves at different scan rates range 10–110 mV/s, and (**b**) display the GCD profile curves at various current densities (0.5 A/g–10 A/g); (**c**) Electrochemical impedance spectroscopy (EIS)-Nyquist plots with Randle’s electrical circuit.

The electrochemical analysis of FMC pure biomass-derived carbon is presented in Figure 8a–c through three commonly used electrochemical measurement techniques: cyclic voltammetry (CV), galvanostatic charge/discharge (GCD), and electrochemical impedance spectroscopy (EIS). These techniques evaluate the electrochemical performance of FMC and FMCA biomass carbon, which operates optimally within a voltage window range of −0.8 V to +0.0 V. Due to the nature of carbon as a negative electrode material, the CV curves and GCD triangular profiles support this observation. The electrochemical properties of pure FMC carbon material data curves and results discussion are shown in Supporting Information’s Figure S1a–c.

Cyclic Voltammetry (CV) analysis was conducted to evaluate the electrochemical properties of activated FMCA carbon shown in Figure 8a. The CV profile was recorded at scan rates ranging from 10 to 110 mV/s to analyze the material’s charge storage behavior. The overall CV curves exhibited a nearly rectangular shape, indicating the absence of faradaic reactions. This suggests that the material follows a pure electric double-layer capacitance (EDLC) mechanism further supporting the absence of redox peaks. A consistent increase in response y-axis current was observed with increasing scan rates, demonstrating good rate capability. The symmetric nature of the CV curves confirms the excellent reversibility of charge storage. The electrode-active FMC carbon material exhibited stable adsorption and desorption of ions, and surface absorption–desorption fast ionic storage (K^+^, OH^−^) for efficient energy storage applications. FMCA carbon materials give good porous active region support to electrochemical behavior, making them suitable for high-performance supercapacitors. These findings highlight the promising potential of activated FMCA carbon for advanced energy storage applications.

The GCD profiles of FMCA materials, as shown in Figure 8b, present the charge–discharge curves, exhibiting a nearly triangular shape characteristic of EDLC behavior. The electrochemical properties of activated FMCA carbon are evaluated through GCD curves recorded at current densities ranging from 0.5 to 10 A/g. The charge–discharge curves indicate that the specific capacitance increases with increasing current density, confirming the efficient ionic absorption–desorption mechanism for energy storage applications. In addition, applied current densities of 0.5 A/g, 1 A/g, 3 A/g, 5 A/g, 7 A/g, and 10 A/g correspond to specific capacitances of 251.17 F/g, 110.52 F/g, 62.77 F/g, 33.76 F/g, 23.20.2 F/g, and 11.62 F/g, respectively. The observed charge storage ability has a good electrochemical response for higher specific capacitance FMCA electrodes. In addition, maximum specific capacitance observed Csp-251.17 at 0.5A/g, for which the enhanced capacitance performance of FMCA is attributed to the activated biomass-derived millet husk carbon, which possesses a highly porous nature, facilitating superior ion transport. The overall GCD curves validate the suitability of FMCA for electrochemical energy storage, emphasizing its high surface area and ionic interaction. These findings highlight the potential of FMCA-based carbon materials for advanced energy storage technologies. Table S2 (from the Supporting Information) presents a comparison of the highest specific capacitance obtained at low current density for biomass-derived activated carbon electrode materials, based on three-electrode analysis and previous literature.

A potentiostatic EIS analysis of the FMCA materials, as shown in Figure 8c, was conducted to find the electrochemical impedance properties over a frequency range of 100 kHz to 1 Hz, with a potential amplitude of 10 mV/s. The Nyquist plot reveals a solution resistance (Rs) of 0.113 Ω and a charge transfer resistance (Rct) of 1.51 Ω, indicating efficient charge transport and minimal resistance at the electrode–electrolyte interface. In addition, Randles electrical circuits consisting of the EIS profile were fitted with BOLOGIC 11.56 version with z-fitting software (EC-LAB V11.50). In the circuit system, the solution resistance in the high-frequency region (Rs), charge-transfer resistance (Rct), and electric double-layer capacitance (Cdl) were respectively combined. In the circuit system, the combined high-frequency region represents the charge transfer characteristics. The relatively low Rct value suggests favorable charge transfer kinetics, making FMCA a promising material for energy storage applications. The nearly vertical tail in the low-frequency region (Warburg Impedance (W_Ω_) reflects excellent capacitive behavior with a leaner plot fit, reinforcing the material’s suitability for EDLC applications. Additionally, the z-fitted values of Randles electrical circuit, Rs, Rct, Cdl, and W_(Ω)_, are provided in Table S2 of the Supporting Information. Overall, the EIS results validate the superior electrochemical characteristics of FMCA, demonstrating its potential for high-performance supercapacitors.

### 4.1. Two-Electrode Symmetry Device Performance

A symmetric two-electrode device was assembled in a pouch-type configuration, where both the materials served as identical anode and cathode electrode components. Subsequently, a Whatman filter paper, soaked in 3M KOH aqueous electrolyte, was placed between the electrodes, with additional electrolyte droplets added inside the pouch case. The device was then sealed using a thin aluminum-laminated polymer film through hot thermal sealing along all four edges. The assembled pouch cell was evaluated in a symmetric two-electrode configuration over a potential range of 0.0 to 1.0 V. Cyclic voltammetry (CV) was performed at various scan rates, and galvanostatic charge–discharge (GCD) testing was conducted to assess electrochemical performance. Additionally, charge-transfer resistance properties were analyzed using potentiostatic electrochemical impedance spectroscopy (EIS), with Nyquist plots recorded for both electrode material configurations: FMC//FMC and FMCA//FMCA.

**Figure 9:**
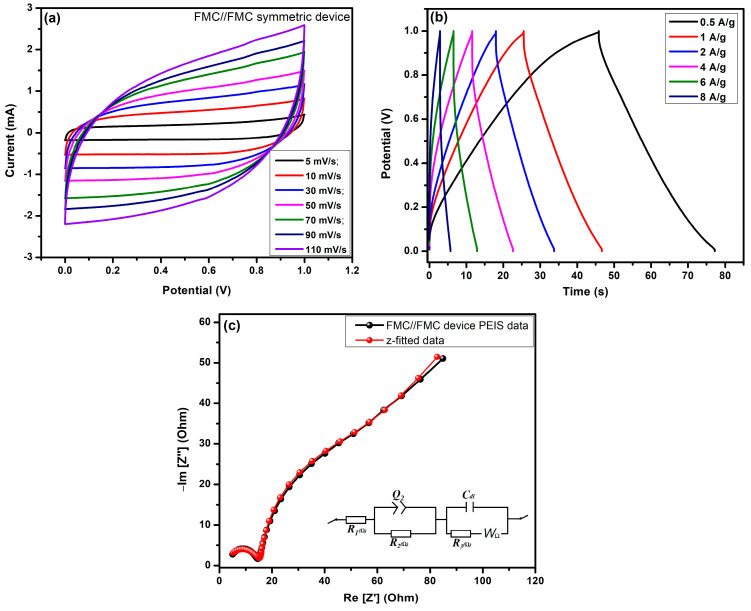
Electrochemical performance of the activated carbon FMC//FMC symmetric devices: (**a**) CV profile at different scan rates, (5–110 mV/s); (**b**) GCD curves of the FMC//FMC symmetric cell at different current densities; (**c**) EIS-Nyquist plots of the device and z-fitted data (inset: Randles electric circuit).

**Figure 10:**
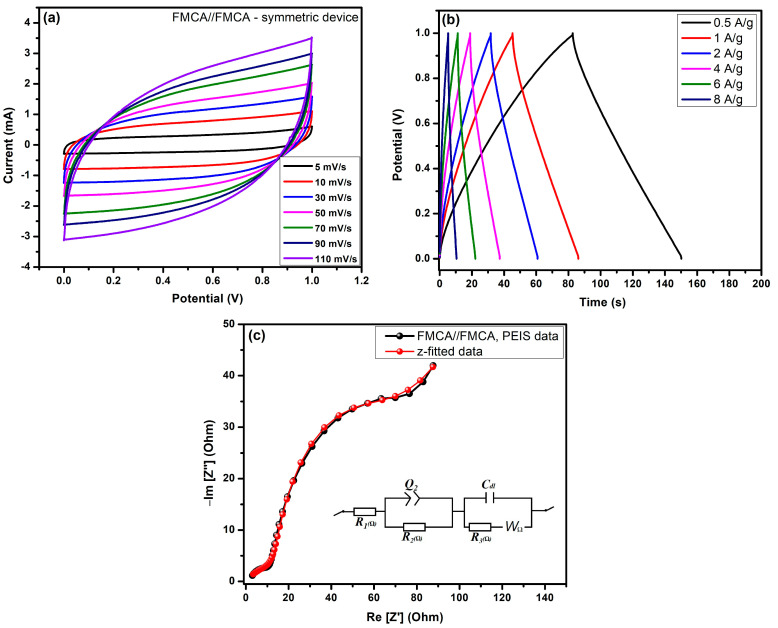
Electrochemical characterization of FMCA//FMCA symmetric supercapacitor devices: (**a**) CV curves recorded at varying scan rates (5–110 mV/s); (**b**) GCD profiles at different applied current densities; (**c**) EIS-Nyquist plots with corresponding Z-fit data (inset: equivalent circuit based on Randles model).

The electrochemical analysis of electrodes using biomass-derived carbon, before and after KOH treatment, was conducted for FMC//FMC and FMCA//FMCA symmetric devices in a two-electrode cell system, as shown in Figure 9a–c and Figure 10a–c.

Cyclic voltammetry (CV) measurements for FMC//FMC and FMCA//FMCA devices, presented in Figure 9a, show that the CV curves exhibit a gradual increase in current (mA) with increasing sweep rates of 5, 10, 30, 50, 70, 90, and 110 mV/s. The FMC//FMC device electrodes demonstrate redox-free behavior, with no visible moisture-related redox humps or Ni-substrate peaks. These CV curve results confirm that they primarily exhibit ideal electric double-layer capacitance (EDLC) behavior. In comparison, in Figure 10a, the FMCA//FMCA symmetric device shows a broader electrochemical window and superior performance. The CV curves of the FMCA//FMCA devices exhibit a broad and wide-range electrochemical response, indicating enhanced charge storage behavior. After KOH activation, the formation of nanoporous structures increases active sites for K^+^ and OH^−^ ion adsorption, improving surface absorption and ion storage. As a result, the FMCA//FMCA device demonstrates superior EDLC-type symmetric supercapacitor performance compared to the FMC//FMC device.

Additionally, the galvanostatic charge–discharge (GCD) profiles of FMC//FMC and FMCA//FMCA active materials, recorded at current densities ranging from 0.5 to 8 A/g, are shown in Figure 8b and Figure 10b. The charge–discharge curves display a triangular shape, characteristic of EDLC behavior, indicating efficient ionic storage through surface adsorption–desorption processes involving K^+^ and OH^−^ ions in pure carbon-based FMC symmetric electrode materials. Figure 9b shows the FMC//FMC device specific capacitance values from equ-1; the calculated and observed values are 55.47 F/g, 34.42 F/g, 22.78 F/g, 16.83 F/g, 12.44 F/g, and 7.03 F/g at current densities of 0.5 A/g, 1 A/g, 2 A/g, 4 A/g, 6 A/g, and 8 A/g, respectively. These results demonstrate good ionic storage capability, making FMC electrodes suitable for energy storage applications. Furthermore, in Figure 10b, the GCD analysis of the FMCA//FMCA symmetric device shows improved specific capacitance values of 82.94 F/g, 48.16 F/g, 33.05 F/g, 21.93 F/g, 15.70 F/g, and 9.21 F/g at the same current densities. This improvement is attributed to KOH activation, which enhances the active surface area and creates a more porous structure, providing a higher number of ionic storage sites in FMCA//FMCA materials.

Electrochemical impedance spectroscopy (EIS) analysis was conducted to evaluate the resistivity of the electrode materials. The Nyquist plot, measured within a frequency range of 100 kHz to 1 Hz with a potential amplitude of 10 mV, is shown in Figure 9 and Figure 10c. In the circuit model, the high-frequency region includes solution resistance (Rs (R_1_), while the charge-transfer resistance (R_2_, R_3_ (Rct)), electric double-layer capacitance (Cdl), and Cdl in the constant-phase element (CPE) (Q) are incorporated. At lower frequencies, the Warburg diffusion coefficient (W_Ω_) accounts for mass transfer effects using the Randles circuit. In the lower frequency region, the plot exhibits a nearly 45-degree Warburg impedance slope. In Figure 10c, the FMC//FMC symmetric device shows a distinct semicircular region in the low-frequency range, with Rs = 1.257 Ω and Rct = 14.92 Ω. In contrast, in Figure 10c, the FMCA//FMCA symmetric device also displays a semicircular region, but with lower resistance values of Rs = 0.89 Ω and Rct = 9.96 Ω. The lower charge transfer resistance (Rct) of the FMCA//FMCA device compared to the untreated FMC electrodes indicates enhanced electrode–electrolyte interface behavior, supporting ideal capacitive characteristics. In the circuit system, the EIS z-fitted parameter values are provided in the Supporting Information: Table S4 for FMC//FMC and Table S5 for FMCA//FMCA symmetric devices.

Finally, in Figure 10a–c, the FMCA//FMCA symmetric device exhibits superior electrochemical performance in CV, GCD, and EIS analyses, demonstrating its high efficiency for energy storage applications. The enhanced capacitance, lower charge transfer resistance, and broad electrochemical window confirm its potential for demonstrating its high efficiency for energy storage applications. Finally, biomass-derived activated carbon exhibits the highest specific capacitance compared to previous literature on biomass-derived activated carbon electrode materials for supercapacitor applications, as shown in Supporting Information Table S6. The reversible absorption–desorption process of ions within the electrode material enhances the electrochemical stability, making FMCA a promising candidate for supercapacitor applications.

### 4.2. Cycling Stability

**Figure 11:**
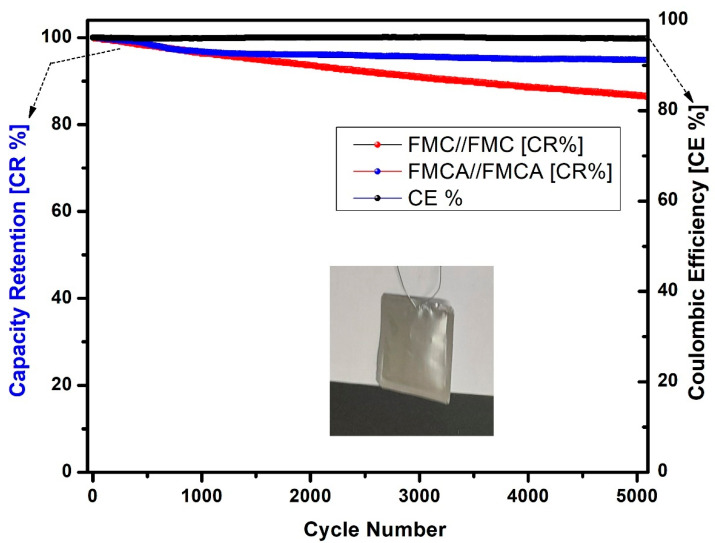
Capacitance retention and coulombic efficiency performance of cycle stability test for the FMC//FMC and FMCA//FMCA symmetric supercapacitor devices (inset: photographic images for FMCA//FMCA symmetric device).

The charge–discharge cycling stability performance is presented in Figure 11, illustrating the capacity retention (CR%) and coulombic efficiency (CE%) of FMC//FMC and FMCA//FMCA symmetric devices over 5000 cycles. The FMC//FMC device demonstrates a capacity retention of 86.82%, whereas the FMCA//FMCA symmetric device exhibits superior performance with a higher capacity retention of 94.89%. Additionally, both devices maintain excellent coulombic efficiency, reaching approximately 99.75%. These results highlight the long-term electrochemical stability of the FMC-activated electrode pouch device, confirming the potential of KOH-activated biomass-derived carbon as a promising material for future energy storage applications. In comparison, the EIS data profile and Nyquist plot after cycling demonstrate that the FMCA//FMCA symmetric device exhibits higher cycling stability with lower resistive properties, even after 5000 cycles. Detailed analysis and discussion of these results are provided in the Supporting Information Figures S2 and S3.

## 5. Conclusions

In this study, thermally activated pure FMC carbon and FMCA carbon materials were successfully developed through KOH activation and carbonization in an argon atmosphere at 800 °C. The biomass-derived activated carbons were synthesized from milled husk and exhibited a well-developed porous surface morphology, as confirmed by FE-SEM and FE-TEM analyses. In addition, electrochemical evaluation demonstrated that the as-prepared FMC and FMCA materials have great promise as electrode materials in symmetric FMCA//FMCA symmetric supercapacitor devices, with a stable operating voltage window from −0.0 V to +1.0 V. The CV curves confirmed an electric double-layer capacitance (EDLC) mechanism, and charge–discharge measurements showed a maximum specific capacitance of 82.94 F/g at 0.5 A/g, along with good stability. Furthermore, the excellent charge storage cycling capability of the FMCA//FMCA-based symmetric device delivered capacity retention CR-94.89% over 5000 cycles. Overall, this study highlights the potential of biomass-derived millet husk carbon as a sustainable and scalable material for future energy storage applications, paving the way for bio-derived carbon materials in high-performance supercapacitors.

## Data Availability

The original contributions presented in this study are included in the article/Supplementary Materials. Further inquiries can be directed to the corresponding authors.

## Supplementary Materials

The following supporting information can be downloaded at: https://www.mdpi.com/article/10.3390/nano15080575/s1. References [52,53,54,55,56] are cited in the Supplementary Materials.
