# Supplementary material for: Biomass-Derived Activated Porous Carbon from Foxtail Millet Husk to Utilizing High-Performance Symmetric Supercapacitor Applications
Source: Nanomaterials (Basel). 2025 Apr 10;15(8):575. doi: 10.3390/nano15080575 (PMC12029297; doi:10.3390/nano15080575)
Supplement: Supplementary file 1 [file nanomaterials-15-00575-s001.zip › nanomaterials-3569537-supplementary.pdf]

Biomass-Derived Activated Porous Carbon from Foxtail Millet Husk to Utilizing High-Performance Symmetric Supercapacitor Applications

Perumal Rajivgandhi [1,†], Vediyappan Thirumal [2,†], Alagan Sekar [1,*] and Jinho Kim [2,*]

[1] Department of Chemistry, Nehru Memorial College (Affiliated to Bharathidasan University),
Puthanampatti, Trichy 621 007, India; rajlibniz@gmail.com
[2] Department of Mechanical Engineering, Yeungnam University,
Gyeongsan-si 38541, Republic of Korea; thirumalvisnu@gmail.com
* Correspondence: alagansek66@gmail.com (A.S.); jinho@ynu.ac.kr (J.K.)
† These authors contributed equally to this work.

Supporting Information (SI)

1.FE-SEM, EDS – elemental mapping

Table S1: SEM, EDS maps for FMCA material element distribution C K, O K, and Si K.

Smart Quant Results			
**Element**	**Weight %**	**Atomic %**	**Error %**
C K	53.57	65.93	8.44
O K	24.24	22.39	8.85
SiK	22.20	11.68	3.08

2. EIS Nyquist z-fitted Parameters

Table S2: EIS-Nyquist equivalent electrical circuit Z fit analysis for FMCA electrode

**Electrical circuit component**	**z-fitted parameters (Rs+Cdl/(Rct+W_{Ω})**

Rs	0.113 Ohm
Cdl	0.003675 F
Rct	1.519 Ohm
$W_{(\Omega)}$	102.6 Ohm.s$^{-1/2}$

2.Electrochemical analysis – (three-electrode system)

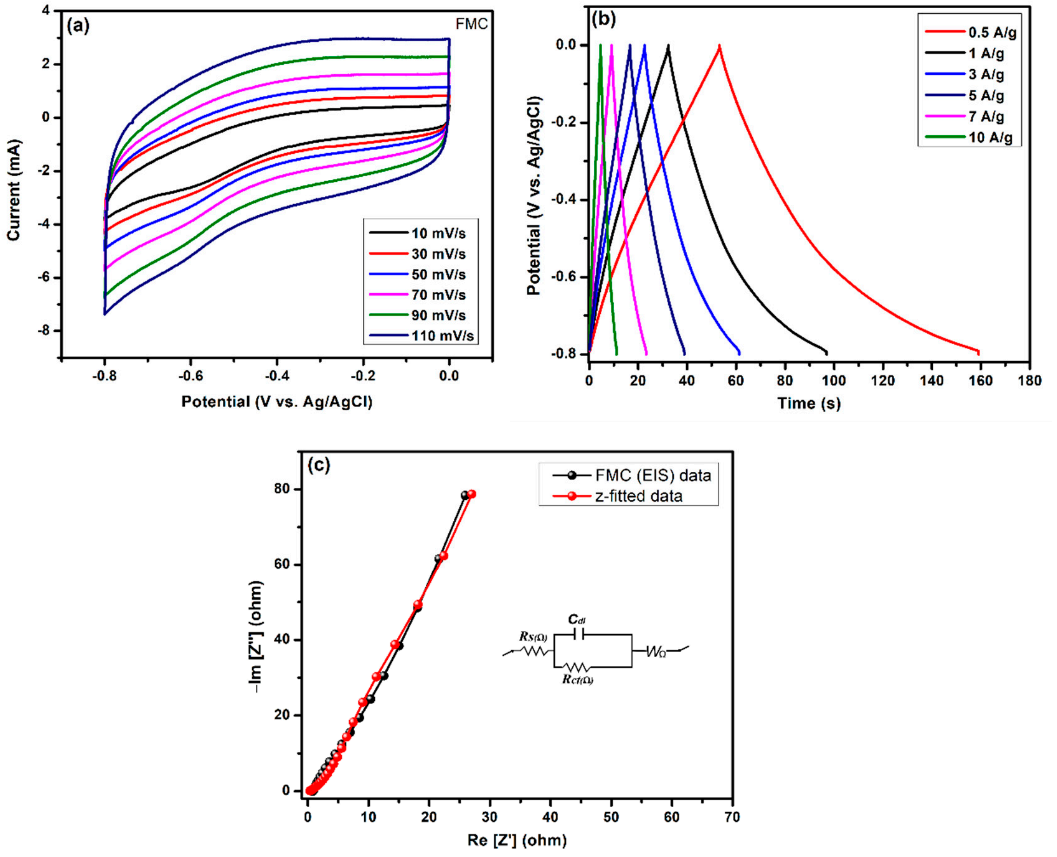

Fig.S1 (a-c) Electrochemical properties of the activated FMC biomass carbon: a CV curves 10 - 110 mV/s; (b) GCD curves with applied current ranges 0.5 – 10 A/g (c) EIS-Nyquist plot with inset Randle's electrical circuit.

Fig. S1 (a) illustrates the cyclic voltammogram (CV) curves of FMC active materials, showing a gradual increase in current with increasing scan rates of 5, 10, 30, 50, 70, 90, and 110 mV/s. The FMC electrode material exhibits redox-free behavior, without noticeable moisture-related redox humps. These results demonstrate that the CV curves primarily reveal electric double-layer capacitance (EDLC) characteristics.

Additionally, the galvanostatic charge-discharge (GCD) profile of FMC active materials, recorded at applied current densities ranging from 0.5 to 10 A/g, is shown in Fig. S1 (b). The charge-discharge curves exhibit a triangular shape, confirming the EDLC nature of FMC carbon, with efficient surface adsorption-desorption processes for fast ionic storage of K^+ and OH^- ions. The specific capacitance values calculated at different current densities are as follows: 106.52 F/g at 0.5 A/g, 65.68 F/g at 1 A/g, 39.90 F/g at 3 A/g, 24.60 F/g at 5 A/g, 17.05 F/g at 7 A/g, and 9.01 F/g at 10 A/g. These findings indicate that FMC electrode materials provide good ionic storage capabilities, making them suitable for energy storage applications.

Furthermore, potentiostatic electrochemical impedance spectroscopy (EIS) analysis was performed to assess the resistivity of the electrode material. The Nyquist plot, measured over a frequency range of 100 kHz to 1 Hz with a potential amplitude of 10 mV, is depicted in Fig. S1 (c). In the circuit model, the high-frequency region solution resistance (Rs), charge-transfer resistance (Rct), and electric double-layer capacitance (Cdl) are incorporated, along with the Warburg diffusion coefficient (W_Ω) at lower frequencies. Additionally, the z-fitted values for Randle's electrical circuit are provided in Table S3. The extracted values from the EIS spectra are Rs = 0.24 Ω and Rct = 1.99 Ω, respectively. The Rct value corresponds to the electrode-electrolyte interface, indicating good capacitive behavior and near-ideal capacitance characteristics. Overall,

the CV, GCD, and EIS results demonstrate the excellent electrochemical performance of FMC pure biomass carbon in a three-electrode half-cell configuration.

Table S3: EIS-Nyquist equivalent electrical circuit z-fitted parameters FMC electrode

**Electrical circuit component**	**z-fitted parameters (Rs+Cdl/(Rct+W_{Ω})**
Rs	0.2425 Ohm
Cdl	0.0005601 F
Rct	1.995 Ohm
$W_{(\Omega)}$	28.52 Ohm.$s^{-1/2}$

3. Symmetric Devices Electrochemical Impedance analysis for Z-fitting parameters

Table S4: EIS - Nyquist plots Z-fitted values with corresponding equivalent circuit based on Randle's model FMC//FMC symmetric devices.

**Electrical circuit component**	**z-fitted parameters: R1+Q2/R2+C3/(R3+W3)**
R1 (R_s)	1.257 Ohm
Q2 (CPE)	0.004653 F.$s^{(a - 1)}$
R2 (R_{ct})	14.926 Ohm
R3 (R_{ct})	35.31 Ohm
C3 (Cdl)	0.0003346 F
W3 ($W_{(\Omega)}$)	79.49 Ohm.$s^{-1/2}$

Table S5: Z-fit data with corresponding equivalent circuit based on Randle's electrical circuit model FMCA//FMCA symmetric devices.

Electrical circuit component	z-fitted parameters: R1+Q2/R2+C3/(R3+W3)
R1 (R_s)	0.894 Ohm
Q2 (CPE)	0.02185 $F.s^{(a - 1)}$
R2 (R_{ct})	9.96 Ohm
R3 (R_{ct})	47.46 Ohm
C3 (Cdl)	0.0003119 F
W3 ($W_{(\Omega)}$)	72.37 $Ohm.s^{-1/2}$

4. EIS data Comparison for Fresh after Cycling data:

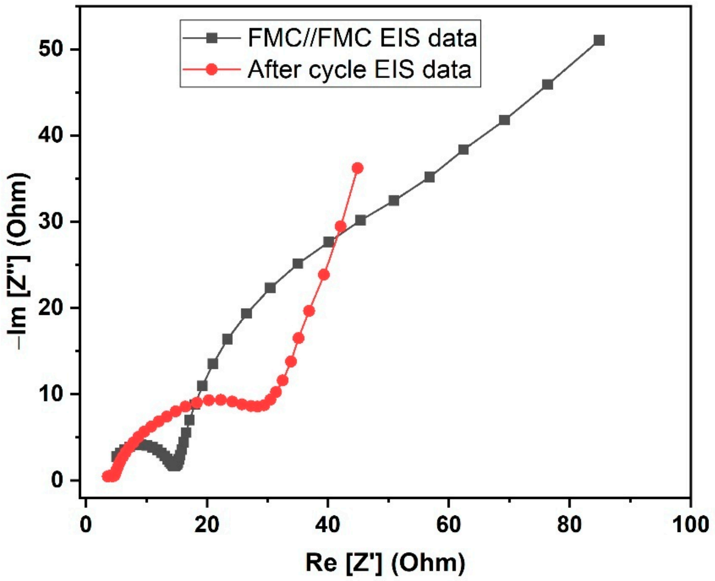

Fig. S2, EIS fresh FMC//FMC symmetric device and after cycling EIS data profile

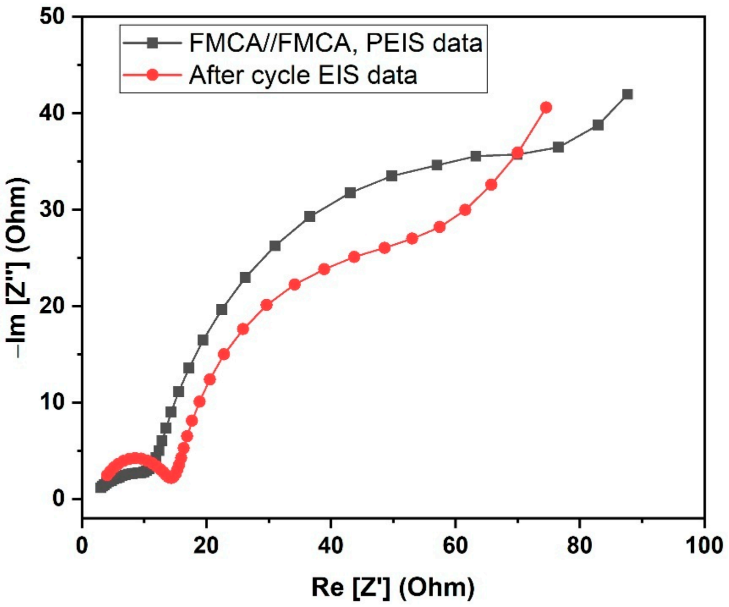

Fig. S3, EIS fresh FMCA//FMCA symmetric device and after cycling EIS data profile

The Nyquist plot of the fresh EIS data for the FMC//FMC symmetric device (Figure S2) illustrates the electrochemical behavior before and after cycling. The EIS curves indicate that the pure FMC carbon exhibits a significant increase in resistive behavior and Warburg impedance after cycling, which affects its capacitive properties.

In comparison, Figure (S3) the FMCA//FMCA device, derived from KOH-activated carbon, demonstrates enhanced capacitive performance due to the presence of more active sites and a porous structure. This activation results in lower resistive changes, even after 5000 cycles, ensuring superior capacitance retention. These findings highlight the potential of activated millet husk-derived biomass carbon as a promising material for future energy storage applications.

Table-S6, The biomass derived activated carbon electrode analysis yielded the highest specific capacitance with applied low current density comparation previous literatures for biomass derived activated carbon electrode materials for supercapacitor applications.

S. No.	Material	Electrolyte	Specific Capacitance (F/g)	Current Density (A/g)	Ref.
1.	Pores Activated Carbon	1 M TEABF4/PC electrolyte	153.66 F/g	0.5 A/g	[1]
2.	Activated carbon (AC) materials Electrodes	Not specified	127.19	0.5 A/g	[2]
3.	Commercial Activated Carbon	1M Na_2SO_4 electrolyte	94 F/g for the	1 A/g	[3]
4.	Ricinus communis shell (RCS) derived activated carbon	3 M KOH	137 F/g	1 A/g	[4]
5.	Biomass tea-waste derived AC	3 M KOH electrolyte	131.95 F/g	0.5 A/g	[5]
**6.**	**Foxtail Millet Husk-derived and activated (FMCA) carbon**	**3 M KOH electrolyte**	**251.17 F/g**	**0.5 A/g**	**This Work**

[1] C.Y. Wu, C.Y. Chang, S.W. Tsai, S.C. Lin, T.C. Hsu, T.H. Hsieh, Activated carbon for supercapacitor electrodes produced by the carbonation and activation of glucose with potassium nitrate, ACS Appl. Energy Mater. 2024, 7, 16, 6873–6886, https://doi.org/10.1021/acsaem.4c00732

[2] Y. Wang, X. Sun, K. Zhang, J. Feng, X. Sun, C. Li, K. Wang, X. Zhang, Y. Ma, Effects of activated carbon types on the CO2 supercapacitive swing adsorption performances. J Solid State Electrochem (2025). https://doi.org/10.1007/s10008-025-06271-8

[3] L. Zhang, Y. Chi, Z. Li, X. Sun, H. Gu, H. Zhang,Y. Chen, G. Z. Chen, Effects of Pore Widening vs oxygenation on capacitance of activated carbon in aqueous sodium sulfate electrolyte, Journal of The Electrochemical Society, 167 (2020) 040524, https://doi.org/10.1149/1945-7111/ab75c8

[4] Rajasekaran, S.J.; Grace, A.N.; Jacob, G.; Alodhayb, A.; Pandiaraj, S.; Raghavan, V. Investigation of Different Aqueous Electrolytes for Biomass-Derived Activated Carbon-Based Supercapacitors. Catalysts, 13 (2023) 286. https://doi.org/10.3390/catal13020286

[5] V. Thirumal, R. Yuvakkumar, G. Ravi, G. Dineshkumar, M. Ganesan, Saad H. Alotaibi, D. Velauthapillai, Characterization of activated biomass carbon from tea leaf for supercapacitor applications, Chemosphere, 291, Part 2 (2022) 132931, https://doi.org/10.1016/j.chemosphere.2021.132931
